# Association of Th2-like inflammation with Tfh/Tph–germinal center immune programmes in submandibular gland lesions of IgG4-related disease

**DOI:** 10.1093/rheumatology/keag394

**Published:** 2026-07-31

**Authors:** Motohisa Yamamoto, Ryuta Kamekura, Masaaki Uehara, Yuta Ichii, Kenichi Takano

**Affiliations:** Department of Rheumatology, Allergy and Clinical Immunology, IMSUT Hospital, The Institute of Medical Science, The University of Tokyo, Tokyo, Japan; Department of Otolaryngology-Head and Neck Surgery, Sapporo Medical University School of Medicine, Sapporo, Japan; Department of Rheumatology, Allergy and Clinical Immunology, IMSUT Hospital, The Institute of Medical Science, The University of Tokyo, Tokyo, Japan; Department of Rheumatology, Allergy and Clinical Immunology, IMSUT Hospital, The Institute of Medical Science, The University of Tokyo, Tokyo, Japan; Division of Cancer Cell Biology, The Institute of Medical Science, The University of Tokyo, Tokyo, Japan; Department of Otolaryngology-Head and Neck Surgery, Sapporo Medical University School of Medicine, Sapporo, Japan

**Keywords:** IgG4-related disease, Th2-like inflammation, Tfh/Tph cells, germinal center, plasmablast

## Abstract

**Objectives:**

IgG4-related disease (IgG4-RD) has long been associated with Th2-predominant immune responses and allergic features. However, the immunological context underlying lesional Th2-like inflammation remains incompletely understood.

**Methods:**

Bulk RNA-seq data from 49 submandibular gland lesions of IgG4-RD were analyzed. Module scores representing Th2, Tfh/Tph, B cell, germinal center B cell (GC_B), plasma cell, macrophage and stromal/fibrotic programmes were calculated. Correlations between Th2 module scores and other immune/stromal modules were assessed using Spearman correlation analysis. Differentially expressed genes between Th2-high and Th2-low lesions were also evaluated.

**Results:**

Th2 module scores were strongly associated with Tfh, Tfh/Tph and Tph helper T cell programmes, as well as with B cell, GC_B and plasma cell signatures. In addition, Th2 module scores positively correlated with M2 macrophage and fibrosis-associated macrophage (FAM) programmes. Differential expression analysis demonstrated increased expression of chronic helper T cell-related genes, including PDCD1, TOX and MAF, together with plasmablast-associated genes such as JCHAIN, MZB1 and PRDM1 in Th2-high lesions. In contrast, serum IgG4 and IgE levels did not significantly differ between Th2-high and Th2-low groups.

**Conclusions:**

Lesional Th2-like inflammation in IgG4-RD was closely associated with chronic Tfh/Tph–germinal center immune programmes linked to plasmablast and M2/FAM macrophage responses. No clear association with available systemic allergy-related parameters was observed in this cohort.

## Graphical abstract

**Graphical Abstract:**
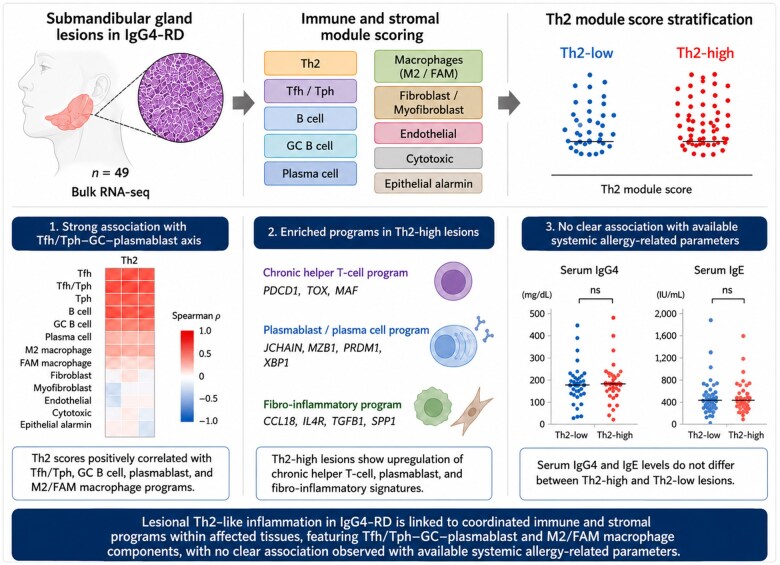

Rheumatology key messages

- Lesional Th2-like inflammation in IgG4-RD was closely associated with chronic Tfh/Tph–germinal center immune programmes rather than with systemic allergic features.
- Th2-high lesions demonstrated coordinated activation of helper T-cell, plasmablast and macrophage/fibro-inflammatory transcriptional programmes within affected tissues.

## Introduction

IgG4-related disease (IgG4-RD) is a systemic fibro-inflammatory disorder characterized by tumefactive lesions, dense lymphoplasmacytic infiltration, storiform fibrosis and elevated serum IgG4 levels [1, 2]. Accumulating evidence has suggested an association between IgG4-RD and Th2-predominant immune responses, based on frequent eosinophilia, elevated serum IgE levels and the relatively high prevalence of allergic diseases in affected patients [3]. Notably, younger patients with IgG4-RD reportedly exhibit allergic comorbidities more frequently than older patients [4], suggesting potential heterogeneity in the immunological background of the disease.

The relationship between IgG4-RD and Th2 immunity has also been supported by studies of IgG4 class-switch recombination. IL-4-producing T follicular helper (Tfh2) cells and related cytokine environments have been implicated in IgG4 class switching, plasmablast differentiation and ectopic germinal center formation in IgG4-RD [5, 6]. In particular, circulating Tfh2 cell expansion has been shown to correlate with serum IgG4 levels, IL-4 levels and plasmablast counts in patients with active IgG4-RD [6]. These observations have led to the widely accepted concept that IgG4-RD represents a Th2-associated immune disorder.

However, despite these findings, the immunological context underlying lesional Th2-like inflammation remains incompletely understood. In particular, it remains unclear whether Th2-like inflammation in affected tissues primarily reflects systemic allergic predisposition or is integrated into local chronic immune programmes associated with germinal center reactions, plasmablast responses and fibro-inflammatory remodelling.

Previous studies have demonstrated associations between circulating Tfh2 cells, IL-4 production, plasmablast expansion and serum IgG4 concentrations in IgG4-RD. However, it remains unclear how Th2-related immune programmes are organized within affected tissues and whether they are linked to broader GC-helper, plasmablast, macrophage and fibro-inflammatory programmes at the lesional level. Addressing this question requires analysis of the transcriptional landscape within diseased organs rather than peripheral immune phenotypes alone.

In the present study, we analyzed bulk RNA-seq data from 49 submandibular gland lesions of IgG4-RD to investigate transcriptionally coordinated immune and stromal programmes associated with lesional Th2-like inflammation. Using a module-based analytical framework, we evaluated the relationships between Th2 programmes and Tfh/Tph-associated helper T cell responses, germinal center activity, plasmablast signatures, macrophage programmes and fibrosis-related stromal responses.

## Methods

### Subjects and tissue samples

This study included submandibular gland tissues from 49 patients with IgG4-related disease (IgG4-RD) collected for diagnostic purposes at Sapporo Medical University. All patients fulfilled the 2020 revised comprehensive diagnostic criteria for IgG4-RD [7]. The study was approved by the Ethics Review Committee of the Institute of Medical Science, The University of Tokyo (Approval No.: 2023-22-0720) and written informed consent was obtained from all participants in accordance with the Declaration of Helsinki.

### Clinical data collection

Clinical and laboratory data were retrospectively collected from medical records. Clinical variables included age, sex, serum IgG4 levels, serum IgE levels, peripheral blood eosinophil counts, disease duration, history of allergic disease and complement levels. All clinical variables were available for analysis, except for one missing value each for C3 and C4.

### RNA sequencing and preprocessing

Total RNA was extracted from submandibular gland tissue using TRIzol reagent (Thermo Fisher Scientific, Waltham, MA, USA). RNA quality was assessed according to the recommendations of the Beijing Genomics Institute (Beijing, China), using OD260/280 ≥ 1.8 and RNA integrity number (RIN) ≥ 7 as quality thresholds.

Library preparation and sequencing were performed using the DNBSEQ-G400 platform (MGI Tech, Shenzhen, China) to generate 100-bp paired-end reads. Transcript quantification was performed using Salmon (version 1.2.1) with protein-coding transcript sequences obtained from GENCODE release 40. Transcript-level abundance estimates were imported into R using the tximport package and summarized at the gene level. Ensembl transcript identifiers were converted to official gene symbols using biomaRt (version 2.50.0).

Gene expression values were normalized as transcripts per million (TPM) and used for subsequent module-based analyses and differential expression analyses. All samples were processed using the same sequencing workflow and platform; therefore, batch correction procedures were not applied.

### Module-based immune architecture analysis

To characterize the immune and stromal architecture associated with lesional Th2-like inflammation, module scores representing predefined immune and stromal programmes were calculated. The analyzed modules included Th2, T follicular helper (Tfh), peripheral helper T cell (Tph), combined Tfh/Tph, B cell, germinal center B cell (GC_B), plasma cell, macrophage, fibrosis-associated macrophage (FAM), fibroblast, myofibroblast, epithelial alarmin, endothelial and cytotoxic lymphocyte signatures.

Representative genes included GATA3, IL4R and CCL18 for the Th2 module; CXCL13, PDCD1, ICOS, IL21 and MAF for the Tfh/Tph module; AICDA and BCL6 for the GC_B module; MZB1, JCHAIN and PRDM1 for the plasma cell module; and CD163, MRC1, SPP1 and CCL18 for macrophage-related modules. A full list of module genes is provided in Supplementary Table S1.

For each module, module scores were calculated as the mean log2-transformed expression of genes included in the corresponding signature.

### Definition of Th2-high and Th2-low lesions

Samples were stratified into Th2-high and Th2-low groups using the median Th2 module score as the cut-off. This dichotomization was introduced primarily as an exploratory approach to facilitate differential expression and clinical comparison analyses. The principal findings of the study were based on continuous correlation analyses between the Th2 module and other immune/stromal programmes. To assess the robustness of the observed associations, additional sensitivity analyses using quartile-based stratification of Th2 module scores were performed.

### Correlation analysis

Spearman rank correlation analysis was performed to evaluate associations between the Th2 module score and other immune or stromal module scores. Multiple testing correction was performed using the Benjamini–Hochberg method.

### Differential expression analysis

Differential gene expression analysis was performed between Th2-high and Th2-low lesions using log2-transformed expression values. Group comparisons for each gene were performed using Welch’s *t* test, followed by Benjamini–Hochberg correction.

### Clinical comparison

Clinical and laboratory parameters were compared between Th2-high and Th2-low groups. Continuous variables were analyzed using nonparametric statistical tests as appropriate, and categorical variables were compared using Fisher’s exact test.

### Statistical analysis and visualization

All analyses were performed using R software (version 4.5.2). Heatmaps, scatter plots, principal component analysis and volcano plots were generated to visualize transcriptionally coordinated immune and stromal programmes and differentially expressed genes. A two-sided *P*-value <0.05 was considered statistically significant.

## Results

### Lesional Th2-like inflammation was associated with Tfh/Tph–GC-related immune programmes in IgG4-RD

To characterize transcriptionally coordinated immune and stromal programmes associated with lesional Th2-like inflammation, we performed a module-based analytical framework using bulk RNA-seq data derived from 49 IgG4-RD submandibular gland lesions.

**Figure 1:**
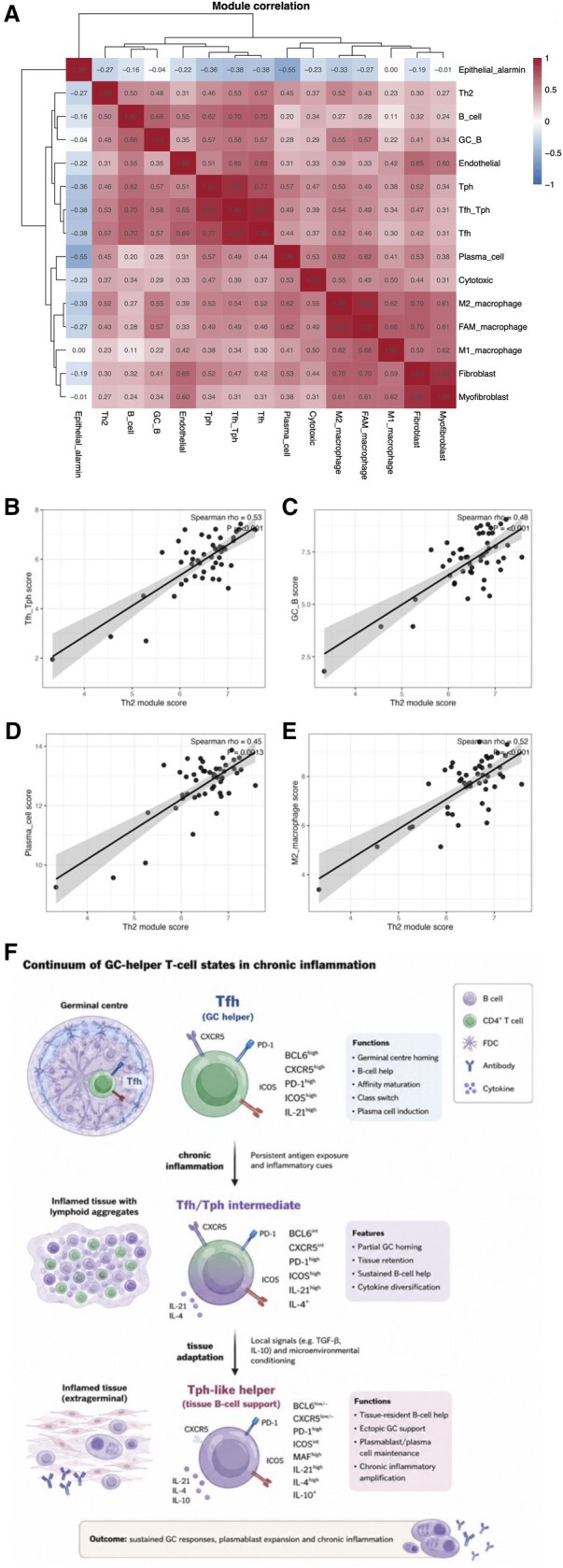
Lesional Th2-like inflammation was associated with GC-helper immune programmes in IgG4-related disease. (**A**) Correlation heatmap showing associations among immune and stromal module scores in IgG4-RD submandibular gland lesions. Positive correlations were observed between the Th2 module and Tfh/Tph-related helper T-cell, germinal center B-cell (GC_B), plasma cell and macrophage-related programmes. (**B**–**E**) Scatter plot analyses demonstrating positive correlations between the Th2 module score and Tfh/Tph score (**B**), GC_B score (**C**), plasma cell score (**D**) and M2 macrophage score (**E**). Spearman correlation coefficients and P values are shown in each panel. (**F**) Proposed transcriptionally inferred immune architecture in IgG4-RD. Th2-like inflammation was associated with chronic Tfh/Tph-related helper T-cell responses, germinal center activation, plasmablast expansion and macrophage-mediated fibro-inflammatory remodelling within affected tissues

Correlation heatmap analysis demonstrated that the Th2 module was positively associated with Tfh, Tfh/Tph, B cell, germinal center B cell (GC_B), plasma cell and M2/FAM macrophage programmes (Fig. 1A). Among these, prominent positive correlations were observed between the Th2 module and Tfh-related helper T cell programmes, including the combined Tfh/Tph module.

Scatter plot analyses further confirmed significant positive correlations between the Th2 module score and Tfh/Tph score (Spearman rho = 0.53, *P* < 0.001), GC_B score (rho = 0.48, *P* < 0.001), plasma cell score (rho = 0.45, *P* = 0.0013) and M2 macrophage score (rho = 0.52, *P* < 0.001; Fig. 1B–E). Additional scatter plot analyses demonstrated similar positive associations between the Th2 module and Tfh, B cell, fibrosis-associated macrophage, fibroblast, endothelial and cytotoxic modules (Supplementary Fig. S1).

Principal component analysis based on immune/stromal module scores demonstrated partial segregation between Th2-high and Th2-low lesions, suggesting coordinated differences in immune and stromal transcriptional programmes associated with lesional Th2-like inflammation (Supplementary Fig. S2).

In contrast, the epithelial alarmin module showed a weak negative correlation with the Th2 module score (rho = −0.27, *P* = 0.063; Supplementary Fig. S1G), suggesting that epithelial-derived allergic alarmin responses may not be the dominant driver of lesional Th2-like inflammation in affected submandibular glands.

Based on these findings, we propose a transcriptionally inferred model in which lesional Th2-like inflammation in IgG4-RD is associated with coordinated Tfh/Tph–germinal center, plasmablast and macrophage-related immune programmes linked to fibro-inflammatory remodelling (Fig. 1F).

### Th2-high lesions exhibited chronic helper T cell- and plasmablast-related transcriptional programmes

To further characterize the molecular features associated with lesional Th2-like inflammation, we performed differential expression analysis between Th2-high and Th2-low lesions.

**Figure 2:**
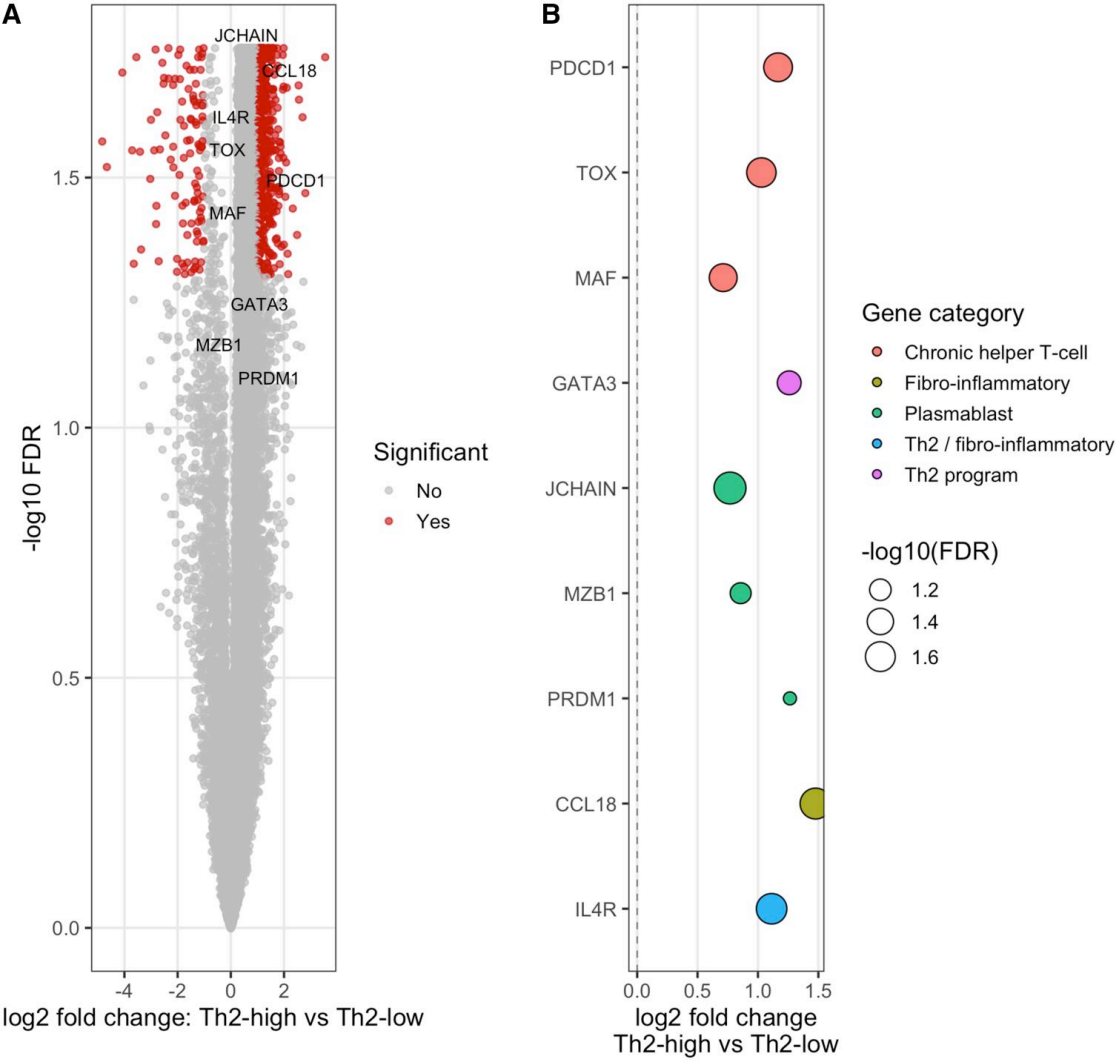
Th2-high lesions exhibited chronic helper T cell- and plasmablast-related transcriptional programmes. (**A**) Volcano plot showing differential gene expression between Th2-high and Th2-low lesions. Selected genes associated with chronic helper T-cell activation, plasmablast differentiation and fibro-inflammatory responses are highlighted. (**B**) Differentially expressed genes associated with Th2-high lesions. Increased expression of chronic helper T-cell-related genes (PDCD1, TOX, MAF, GATA3), plasmablast-associated genes (JCHAIN, MZB1, PRDM1) and fibro-inflammatory genes (CCL18, IL4R) was observed in Th2-high lesions

Volcano plot analysis demonstrated distinct transcriptional programmes between Th2-high and Th2-low lesions (Fig. 2A). Th2-high lesions demonstrated increased expression of chronic helper T cell-related genes, including PDCD1, TOX, MAF and GATA3, together with plasmablast-associated genes such as JCHAIN, MZB1 and PRDM1 (Fig. 2B and Supplementary Table S2). In addition, CCL18 and IL4R expression were elevated in Th2-high lesions, supporting the association between Th2-like inflammation and chronic fibro-inflammatory immune activation.

Interestingly, canonical epithelial alarmin-related pathways were not prominently enriched, consistent with the weak inverse association observed between epithelial alarmin and Th2 module scores (Supplementary Fig. S1G).

### Clinical characteristics of Th2-high and Th2-low lesions

Patients were stratified into Th2-high and Th2-low groups according to the median Th2 module score. Clinical characteristics of the two groups are summarized in Table 1.

**Table 1:** Clinical characteristics of patients with Th2-high and Th2-low IgG4-RD submandibular gland lesions.

	Th2-high	Th2-low	*P* value
Number of patients	25	24	
Age, years	67 (59–70)	63 (56–69)	0.302
Male sex, *n* (%)	36.0	58.3	0.123
Serum IgG4, mg/dl	397 (287–948)	359 (244–783)	0.910
Serum IgE, IU/ml	306 (93–621)	254 (94–686)	0.551
Eosinophil count,/μl	216 (114–311)	194 (85–304)	0.531
CRP, mg/dL	0.10 (0.07–0.10)	0.10 (0.10–0.13)	0.114
Complement (C3), mg/dl	106 (88–113)	96 (83.5–112)	0.699
Complement (C4), mg/dl	21 (14–26)	21 (13.5–25.5)	0.897
Allergic comorbidity, *n* (%)	20.0	29.2	0.467
Number of involved organs	2.6	2.6	0.926

Values are presented as median (interquartile range) or number (%), as appropriate.

Patients were stratified into Th2-high and Th2-low groups according to the median Th2 module score.

Continuous variables were compared using nonparametric statistical tests, and categorical variables were compared using Fisher’s exact test.

Serum IgG4, serum IgE, eosinophil counts and allergic history were available for all patients. C3 and C4 values were unavailable in one patient each.

To further assess the robustness of the observed associations, we performed sensitivity analyses using quartile-based stratification of Th2 module scores. Comparisons between the highest (Q4) and lowest (Q1) quartiles yielded findings consistent with those obtained using median-based stratification, including enrichment of Tfh/Tph, GC_B, plasma cell, M2 macrophage and FAM macrophage programmes in Th2-enriched lesions (Supplementary Fig. S3). These findings support the stability of the observed associations across alternative stratification approaches.

No significant differences were observed in serum IgG4 levels, serum IgE levels, eosinophil counts, allergic comorbidities, or major demographic characteristics between Th2-high and Th2-low lesions.

These findings suggest that, in this cohort, lesional Th2-like immune programmes were more closely associated with coordinated local immune and stromal transcriptional programmes than with available systemic allergy-related parameters.

## Discussion

Our findings extend previous observations linking circulating Tfh2 cells, IL-4, plasmablasts and serum IgG4 levels in IgG4-RD. Rather than focusing on individual immune cell populations or circulating biomarkers, the present study examined coordinated transcriptional programmes within affected tissues. In doing so, we identified a consistent association between lesional Th2-like inflammation and Tfh/Tph-, GC_B-, plasmablast- and macrophage-related programmes, suggesting a broader tissue-level organization of chronic immune responses in IgG4-RD.

In this study, we investigated the immunological context of lesional Th2-like inflammation in IgG4-RD using bulk RNA-seq data from 49 submandibular gland lesions. The main finding was that Th2-like inflammatory programmes were closely associated with Tfh/Tph-related helper T cell programmes, germinal center B cell signatures, plasma cell programmes and M2/FAM macrophage signatures. These results suggest that lesional Th2-like inflammation in IgG4-RD may not exist as an isolated allergic-type response but rather may be embedded within a coordinated GC-helper immune architecture inferred from co-enriched transcriptional programmes.

IgG4-RD has long been considered a Th2-associated disease because of frequent eosinophilia, elevated serum IgE, allergic comorbidities and the biological link between IL-4-producing helper T cell responses and IgG4 class switching. Previous studies have shown that circulating Tfh2 cells are increased in IgG4-RD and correlate with serum IgG4, IL-4 levels and plasmablast numbers, supporting a role for Tfh2-mediated B cell help in disease pathogenesis [6]. Our findings extend this concept by showing that, within affected submandibular gland tissue, Th2-like signatures are transcriptionally linked to Tfh/Tph, GC_B and plasma cell modules. Thus, the Th2-like programme observed in IgG4-RD lesions may reflect a coordinated helper T cell transcriptional programme associated with GC_B and plasma cell signatures within affected tissues. However, the cellular mechanisms underlying these local Th2-like transcriptional programmes remain incompletely understood. In particular, the extent to which Th2-associated and Tfh/Tph-associated signatures arise from overlapping or distinct cellular populations cannot be determined from bulk RNA-seq data alone.

An important observation was that Th2-high lesions showed increased expression of PDCD1, TOX, MAF and GATA3, together with plasmablast-associated genes such as JCHAIN, MZB1 and PRDM1. This pattern is consistent with a chronic helper T cell state coupled to antibody-producing cell programmes. Previous reviews have emphasized the potential interplay between Tfh and Tph cells in IgG4-RD pathogenesis, particularly in the context of chronic inflammation and ectopic lymphoid responses [8]. Our data support this concept at the lesional transcriptomic level and suggest that Tfh/Tph-like helper programmes may provide an immunological bridge between Th2-like inflammation and local B cell/plasmablast responses.

We also found that Th2 module scores were positively associated with M2 and FAM macrophage signatures. This finding is relevant because IgG4-RD is characterized not only by lymphoplasmacytic inflammation but also by fibro-inflammatory tissue remodelling. The association between Th2-like programmes and macrophage/fibrotic modules suggests that helper T cell–B cell immunity and fibro-inflammatory remodelling may represent coordinated transcriptional programmes within affected tissues. Therefore, lesional Th2-like inflammation may contribute to, or coexist with, macrophage-mediated fibrotic remodelling rather than representing a purely humoral or allergic phenomenon.

Notably, serum IgG4 and IgE levels, eosinophil counts and allergic comorbidities did not significantly differ between Th2-high and Th2-low lesions. This result should be interpreted cautiously. Although no clear association with available systemic allergy-related parameters was observed in this cohort, the relationship between systemic allergic status and tissue-level immune organization remains incompletely understood. IgG4-RD is known to be associated with allergic diseases and atopy in a subset of patients, particularly in head, neck and thoracic involvement; however, the relationship between systemic allergic status and tissue-level immune organization remains incompletely understood [9]. Our findings suggest that lesional Th2-like inflammation in submandibular glands may be more closely related to local GC-helper and fibro-inflammatory immune programmes than to circulating IgE or clinical allergy alone.

The epithelial alarmin module showed only a weak inverse association with the Th2 module. This does not exclude the contribution of allergic predisposition or mucosal alarmin responses in IgG4-RD, particularly because our analysis was limited to submandibular gland tissue rather than airway or nasal mucosa. However, within the affected salivary gland lesions analyzed here, epithelial-derived alarmin signatures did not appear to be the dominant correlate of Th2-like inflammation. This supports a model in which local Th2-like inflammation is associated with coordinated helper T cell and B cell transcriptional programmes within affected tissues.

Importantly, the principal conclusions of the present study were derived from continuous correlation analyses rather than binary classification. Although median-based stratification was used to facilitate differential expression and clinical comparison analyses, additional quartile-based sensitivity analyses yielded findings consistent with the primary analyses, supporting the robustness of the observed associations across alternative stratification approaches.

This study has several limitations. First, the analysis was based on bulk RNA-seq, which does not allow direct identification of the cellular source of each transcript. Second, the study focused on submandibular gland lesions, and the findings may not be generalizable to all organs involved in IgG4-RD. Third, spatial relationships among Tfh/Tph-like cells, GC_B cells, plasmablasts, macrophages and fibroblasts could not be directly assessed. Therefore, the proposed framework should be interpreted as a transcriptionally inferred model rather than direct evidence of cellular interaction, lineage relationships, or spatial organization within lesional tissues. Furthermore, the mechanisms driving local Th2-like transcriptional programmes could not be resolved using bulk RNA-seq alone. In particular, whether Th2-associated and Tfh/Tph-associated signatures arise from overlapping or distinct cellular populations remains unclear. Future studies using single-cell and spatial transcriptomic approaches will be required to validate the proposed transcriptional framework and clarify the cellular and spatial relationships underlying these coordinated immune programmes, as well as their relationship to fibrosis and treatment response.

In addition, although no clear association with available systemic allergy-related parameters was observed in this cohort, the relatively modest sample size may have limited the ability to detect weaker associations between local Th2-like inflammation and systemic allergic status. Furthermore, paired peripheral blood transcriptomic data were not available for the patients included in this study. Consequently, direct comparisons between local tissue immune programmes and systemic immune transcriptional responses could not be performed. The present conclusions regarding systemic allergic status are therefore based on available clinical parameters, including serum IgG4 levels, serum IgE levels, eosinophil counts and allergic comorbidities, rather than transcriptomic analyses of peripheral blood. Future studies integrating paired tissue and blood transcriptomic profiling will be important to clarify the relationship between local tissue immune programmes and systemic immune responses in IgG4-RD.

In conclusion, our findings suggest that Th2-like inflammation in IgG4-RD submandibular gland lesions is closely associated with coordinated Tfh/Tph–germinal center, plasmablast and macrophage/fibrotic transcriptional programmes. By examining transcriptional programmes within affected tissues rather than peripheral immune phenotypes alone, this study provides a tissue-based perspective on Th2-like inflammation in IgG4-RD and extends previous observations linking Tfh2 cells, IL-4, plasmablasts and IgG4 production. These results suggest that lesional Th2-like inflammation is associated with coordinated fibro-inflammatory immune programmes within affected tissues. Although no clear association with available systemic allergy-related parameters was observed in this cohort, the present findings should not be interpreted as evidence of a definitive dissociation between local Th2-like inflammation and systemic allergic responses. Rather, they indicate that no clear association was detectable using the available clinical parameters examined in this study.

## Supplementary Material

keag394_Supplementary_Data

## Data Availability

The bulk RNA sequencing data generated in this study have been deposited in the NCBI Sequence Read Archive (SRA) under BioProject accession number PRJNA1482328.
