# Supplementary material for: Association of Th2-like inflammation with Tfh/Tph–germinal center immune programmes in submandibular gland lesions of IgG4-related disease
Source: Rheumatology (Oxford). 2026 Jul 31;65(8):keag394. doi: 10.1093/rheumatology/keag394 (PMC13457081; doi:10.1093/rheumatology/keag394)
Supplement: Supplementary file 1 [file keag394_supplementary_data.docx]

**Supplementary Figure S1. Additional scatter plot analyses between the Th2 module and immune/stromal programs.**

Scatter plot analyses demonstrating associations between the Th2 module score and additional immune/stromal module scores, including Tfh, B-cell, fibrosis-associated macrophage (FAM), fibroblast, endothelial, cytotoxic, and epithelial alarmin modules.

**Supplementary Figure S2. Principal component analysis of immune/stromal module scores.**

Principal component analysis (PCA) based on immune and stromal module scores in IgG4-RD submandibular gland lesions. Partial segregation between Th2-high and Th2-low lesions was observed, suggesting coordinated differences in immune and stromal transcriptional programs associated with lesional Th2-like inflammation.

**Supplementary Figure S3. Sensitivity analysis using quartile-based stratification of Th2 module scores.**

Samples were stratified according to quartiles of Th2 module scores. Comparisons between the highest (Q4) and lowest (Q1) quartiles demonstrated significantly higher Tfh/Tph, GC_B, plasma cell, M2 macrophage, and FAM macrophage module scores in Th2-enriched lesions, supporting the robustness of the observed associations across alternative stratification approaches.

**Supplementary Table S1. Gene signatures used for immune and stromal module analysis.**

| Module | Gene | Status |
| --- | --- | --- |
| Th2 | GATA3, IL4R, IL13RA1, CCR4, PTGDR2, CCL17, CCL18, CCL22, STAT6 | present |
| Tfh_Tph | CXCL13, PDCD1, ICOS, IL21, MAF, BCL6, CXCR5, SLAMF6, CD200 | present |
| Tfh | CXCR5, PDCD1, BCL6, ICOS, IL21, SLAMF6 | present |
| Tph | PDCD1, CXCL13, IL21, MAF, ICOS | present |
| B_cell | MS4A1, CD79A, CD79B, CD19, CD22, BANK1 | present |
| GC_B | AICDA, BCL6, RGS13, MEF2B, MKI67, STMN1 | present |
| Plasma_cell | MZB1, XBP1, JCHAIN, SDC1, PRDM1, IGHG4 | present |
| M1_macrophage | IL1B, TNF, CXCL9, CXCL10, NOS2 | present |
| M2_macrophage | CD163, MRC1, MSR1, IL10, TGFB1, CCL18 | present |
| FAM_macrophage | SPP1, CCL18, CD163, MRC1, TGFB1 | present |
| Fibroblast | COL1A1, COL1A2, COL3A1, DCN, LUM, PDGFRA, PDGFRB, POSTN | present |
| Myofibroblast | ACTA2, TAGLN, MYL9, COL1A1, COL3A1, POSTN | present |
| Epithelial_alarmin | IL33, TSLP, IL25, AREG, KRT8, KRT18, KRT19 | present |
| Endothelial | PECAM1, VWF, CDH5, KDR | present |
| Cytotoxic | GZMB, GZMA, PRF1, NKG7, GNLY | present |
|  |  |  |

List of genes used to calculate immune and stromal module scores.

**Supplementary Table S2. Differentially expressed genes between Th2-high and Th2-low lesions.**

| **Gene** | **Category** | **logFC** | **p_value** | **FDR** | **Direction** |
| --- | --- | --- | --- | --- | --- |
| TOX | Chronic helper T-cell | 1.028 | 0.00265 | 0.027 | Up in Th2-high |
| PDCD1 | Chronic helper T-cell | 1.167 | 0.00353 | 0.029 | Up in Th2-high |
| MAF | Chronic helper T-cell | 0.712 | 0.00446 | 0.033 | Up in Th2-high |
| CCL18 | Fibro-inflammatory | 1.477 | 0.00112 | 0.021 | Up in Th2-high |
| TGFB1 | Fibro-inflammatory | 0.934 | 0.00194 | 0.024 | Up in Th2-high |
| SPP1 | Fibro-inflammatory | 0.317 | 0.638 | 0.768 | Up in Th2-high |
| JCHAIN | Plasmablast | 0.768 | 0.00033 | 0.018 | Up in Th2-high |
| MZB1 | Plasmablast | 0.856 | 0.016 | 0.066 | Up in Th2-high |
| PRDM1 | Plasmablast | 1.265 | 0.0245 | 0.087 | Up in Th2-high |
| XBP1 | Plasmablast | -0.319 | 0.0354 | 0.111 | Down in Th2-high |
| BCL6 | Tfh/GC program | 0.747 | 0.16 | 0.317 | Up in Th2-high |
| ICOS | Tfh/Tph program | 1.236 | 0.0518 | 0.144 | Up in Th2-high |
| CXCL13 | Tfh/Tph program | 1.049 | 0.153 | 0.307 | Up in Th2-high |
| IL21 | Tfh/Tph program | 0.174 | 0.298 | 0.484 | Up in Th2-high |
| IL4R | Th2 / fibro-inflammatory | 1.113 | 0.00163 | 0.023 | Up in Th2-high |
| GATA3 | Th2 program | 1.259 | 0.0108 | 0.052 | Up in Th2-high |

Selected genes associated with chronic helper T-cell programs, plasmablast differentiation, and fibro-inflammatory responses in Th2-high lesions.
